# Phosphoinositide 3 Kinase γ Plays a Critical Role in Acute Kidney Injury

**DOI:** 10.3390/cells11050772

**Published:** 2022-02-23

**Authors:** Xiaogao Jin, Qinjun Chu, Liwei Sun, Melanie Tran, Yanlin Wang

**Affiliations:** 1 Department of Anesthesiology and Perioperative Medicine, Zhengzhou Central Hospital Affiliated to Zhengzhou University, Zhengzhou 450007, China; jimmynetchu@163.com (Q.C.); mzk123456@zzu.edu.cn (L.S.); 2 Research of Trauma Center, Zhengzhou Central Hospital Affiliated to Zhengzhou University, Zhengzhou 450007, China; 3 Center for Advanced Medicine, College of Medicine, Zhengzhou University, Zhengzhou 450007, China; 4 Section of Nephrology, Department of Medicine, Baylor College of Medicine, Houston, TX 77030, USA; 5 Division of Nephrology, Department of Medicine and Department of Cell Biology, University of Connecticut School of Medicine, Farmington, CT 06030, USA; metran@uchc.edu

**Keywords:** PI3Kγ, acute kidney injury, inflammation, ischemia-reperfusion injury

## Abstract

Inflammatory cells contribute to the pathogenesis of renal ischemia-reperfusion injury (IRI). However, the signaling mechanisms underlying the infiltration of inflammatory cells into the kidney are not well understood. In this study, we examined the effects of phosphoinositide 3 kinase γ (PI3Kγ) on inflammatory cells infiltration into the kidney in response to ischemia-reperfusion injury. Compared with wild-type mice, PI3Kγ knockout mice displayed less IRI in the kidney with fewer tubular apoptotic cell. Furthermore, PI3Kγ deficiency decreased the number of infiltrated neutrophils, macrophages, and T cells in the kidney, which was accompanied by a decrease in the expression of pro-inflammatory cytokines in the kidney. Moreover, wild-type mice treated with AS-605240, a selective PI3Kγ inhibitor, displayed less tubular damage, accumulated fewer inflammatory cells, and expressed less proinflammatory molecules in the kidney following IRI. These results demonstrate that PI3Kγ has a critical role in the pathogenesis of kidney damage in IRI, indicating that PI3Kγ inhibition may serve as a potential therapeutic strategy for the prevention of ischemia-reperfusion-induced kidney injury.

## 1. Introduction

Acute kidney injury (AKI) is a common clinical disorder that can cause high morbidity and mortality. Ischemia-reperfusion injury (IRI) is a common risk of AKI [1,2,3]. It is well known that inflammatory cells can infiltrate the kidney and cause damage after IRI [3]. Therefore, it is critical to understand the pathogenic mechanisms of IRI so that effective therapy can be developed.

IRI not only causes damage to the kidney epithelial cells directly by hypoxia, but also leads to the infiltration of inflammatory cells after ischemia. This results in damage to the epithelial cells through the production of pro-inflammatory molecules, such as IL-1, TNF-alpha, MPC, IL-6 and adiponectin [3,4]. These cytokines can induce apoptosis in the epithelial cells [5,6]. To reveal the mechanism of AKI in IRI, it is important to dissect the signaling pathways leading to the infiltration of inflammatory cells into the kidney after IRI. In our previous study, we demonstrated that the PI3Ks inhibitor could block the migration of macrophages induced by adiponectin in vitro, which contributed to the infiltration of inflammatory cells in the kidney with IRI [3]. These results suggest that PI3Ks may take part in the mechanism of kidney damage after IRI.

The phosphoinositide 3-kinase (PI3K) family takes part in the regulation of a diverse array of cellular functions such as cell movement, glucose transport, mitogenesis, and cell survival [7,8]. The PI3Ks are divided into three groups, classes I, II and III, based on their domain structure, amino acid sequence, substrate specificity, and mode of regulation [7]. PI3Kγ belongs to the class I group and is thought to have a central role in the chemotactic function of macrophages and neutrophils [9]. PI3Kγ is a heterodimer consisting of p110c plus a regulatory subunit of p84 or p101, which is regulated by G-protein subunits after the activation of G-protein-coupled receptors (GPCRs) [10]. The 3′ position of the inositol ring of phosphatidylinositol 4,5-bisphosphate (PtdIns(4,5) P2) could be phosphorylated to become PtdIns(3,4,5)P3. PtdIns(3,4,5)P3 contributes to the combination of the molecules with a pleckstrin homology (PH) domain to the membrane [11]. The chemotactic responses may be triggered by protein kinase B (PKB), which combines PtdIns(3,4,5)P3 and filamentous actin at the leading edge of the cell. The internal PtdIns(3,4,5)P3 gradient produced by Rho family GTPases enhances the movement function after the combination of PtdIns(3,4,5)P3 and filamentous actin [11]. PI3Kγ is found in many cell types, but it is the most abundant in myeloid cells, particularly neutrophils and macrophages. The role of PI3Kγ in the pathogenesis of kidney injury after IRI is not known.

In this study, we examined the role of PI3Kγ in the infiltration of inflammatory cells into the kidney and its contribution to AKI after IRI using PI3Kγ knockout (KO) mice and a PI3Kγ inhibitor. Our results demonstrate that genetic knockout or pharmacological inhibition of PI3Kγ reduces kidney inflammation and tubular epithelial cell apoptosis, thereby preventing the kidney from IRI. 

## 2. Materials and Methods

### 2.1. Animals

The Guidelines of Laboratory Animal Care were strictly followed in animal experiments. The Institutional Animal Care and Use Committee of the Baylor College of Medicine approved the protocol for the animal experiments before the study. Wild-type (WT) C57BL/6 mice were commercially obtained from the Jackson Laboratory (Bar Harbor, ME). The PI3Kγ-KO mice on a C57BL/6 background were produced by replacement of the first three exon of PI_3_Kγ with the sequence of green fluorescent protein (GFP) [12,13]. Male WT mice and PI3Kγ-KO mice (8–12 weeks old and weighing 20–30g) were used for the experiments. IRI was carried out as previously reported [3,14]. Briefly, anesthesia was performed by intraperitoneal injection of the combination of ketamine (80 mg/kg) and xylazine (10 mg/kg). Kidneys were exposed and subjected to ischemia by clamping renal pedicles using non-traumatic micro-aneurism clamps. The clamps were removed after 30 min of ischemia. The mice were kept warm on a heat pad throughout the procedure. Sham-control mice had the same surgical procedure except pedicle clamping. Mice were sacrificed 24 h after reperfusion and then the kidneys were harvested for Western blot analysis, immunohistochemistry (IHC), and RT-PCR. For pharmacological study, AS-605240 (30 mg/kg) was administrated intraperitoneally 4 h before IRI in WT mice.

### 2.2. Kidney Function

Serum creatinine was measured using a commercially available creatinine assay kit (BioAssay Systems, Hayward, CA, USA). Blood urea nitrogen (BUN) was measured fluorometrically as previously described [3,14].

### 2.3. Immunohistochemistry

Immunohistochemistry was carried out on paraffin-embedded kidney sections [15]. Antigen unmasking solution (Vector Laboratories, Burlingame, CA, USA) was used to retrieve the antigen after fixation. Three percent H_2_O_2_ was used to block endogenous peroxidase activity before blocking. After incubation with 5% normal serum, slides were incubated with primary antibodies in a humidified chamber overnight. After washing, kidney sections were incubated with secondary antibodies, followed by ABC solution as previously reported [16]. Diaminobenzidine solution was applied to visualize the secondary antibody conjugated with horseradish peroxidase (HRP). Red substrate was used to visualize the secondary antibody conjugated with alkaline phosphatase (AP). Hematoxylin was used for nuclear staining. The sections were dehydrated with ethanol, cleared with histoclear, and mounted with mounting medium. NIS Element software (Nikon Instruments, Melville, NY, USA) with Nikon microscope image system (Nikon Instruments) were utilized to quantify the protein expression levels in the kidney.

### 2.4. Apoptosis Detection

ApopTag plus Peroxidase in situ Apoptosis Detection Kit (Millipore, Billerica, MA, USA) was used to detect apoptosis [17,18]. The apoptotic cells were determined by a terminal transferase dUTP nick-end labeling (TUNEL) assay. The numbers of TUNEL-positive cells per high-power field were quantified in a blinded manner.

### 2.5. Quantitative Real-Time Reverse Transcriptase PCR

TRIzol reagent (Invitrogen, Carlsbad, CA) was used to extract total RNA from kidney tissues [19,20]. RNA (1 µg) was reverse transcribed into cDNA by SuperScript II reverse transcriptase. IQ SYBR green supermix reagent (Bio-Rad, Herculus, CA) was used for Realtime PCR in a Bio-Rad real-time PCR machine. Gene expression was quantified using the comparative Ct method (ΔΔCt). The relative quantification was presented as 2^-ΔΔCt^. The ratios of the genes of interest over glyceraldehyde-3-phosphate dehydrogenase (GAPDH) were analyzed. The primer sequences included: IL-6-forward, 5-AGGATACCACTCCCAACAGACCTG-3, reverse, 5-CTGCAAGTGCATCATCGTTGTTCA-3; TNFα-forward, 5-CATGAGCACAGAAAGCATGATCCG-3, reverse, 5-AAGCAGGAATGAGAAGAGGCTGAG-3; MCP-1-forward, 5-TCACCTGCTGCTACTCATTCACCA-3, reverse, 5-TACAGCTTCTTTGGGACACCTGCT-3; MIP-2-forward, 5-AAAGTTTGCCTTGACCCTGAAGCC-3, reverse, 5-TCCAGGTCAGTTAGCCTTGCCTTT-3; and GAPDH-forward, 5-CCAATGTGTCCGTCGCGTGGATCT-3, reverse, 5-GTTGAAGTCGCAGGAGACAACC-3.

### 2.6. Western Blot Analysis

Proteins were extracted using RIPA buffer containing a cocktail of protease inhibitors. The extracted protein concentration was determined by a Bio-Rad protein assay [21,22]. Equal amounts of protein were loaded onto SDS-polycrylamide gels in a Tris/SDS buffer system. The separated proteins on gels were then transferred onto nitrocellulose membranes. After blocking, the membranes were incubated with primary antibodies overnight. After washing, the membranes were incubated with the fluorescence-conjugated secondary antibodies. The proteins were detected using an Odyssey (LI-COR Bioscience, Lincoln, NE, USA) IR scanner. NIH Image/J software (National Institutes of Health, Bethesda, MD, USA) was used to quantify the protein expression.

### 2.7. Statistical Analysis

All data were expressed as mean ± SD. ANOVA was used to compare multiple groups followed by the Bonferroni procedure for a comparison of the means. Tubular damage scores were analyzed by the Wilcoxon rank sum test. *p* < 0.05 was considered statistically significant.

## 3. Results

### 3.1. PI3kγ Deficiency or Inhibition Protects the Kidney from IRI

**Figure 1:**
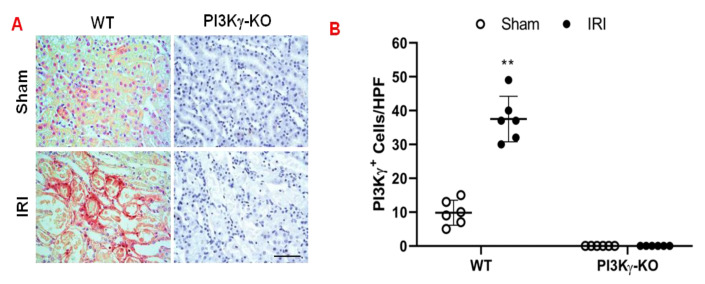
PI_3_Kγ expressed in the kidney in the mice after IRI. (**A**) Representative photomicrographs of PI_3_Kγ (red) expression in kidney slides, which were counterstained with hematoxylin (blue). (**B**) Quantitative analysis of PI3Kγ positive cells in the kidneys of PI3Kγ-KO and WT mice. ** *p* < 0.01 vs. WT sham. *n* = 6 per group. Mean ± SD is 9.83 ± 3.71 for Sham in WT, 37.50 ± 6.72 for IRI in WT, 0.33 ± 0.82 for Sham in PI3Kγ, and 0 ± 0 for IRI in PI3Kγ. HPF means high power field.

To examine the contribution of PI3Kγ to the development of acute kidney injury, we subjected WT and PI3Kγ KO mice to 30 min ischemia followed by 24 h reperfusion. The number of PI3kγ-positive cells in the kidney of WT mice increased significantly after IRI when compared with sham-operated WT mice (Figure 1A,B). In contrast, PI3Kγ-positive staining in the kidney of KO mice was absent in both sham and IRI groups indicating PI3Kγ was disrupted in the KO mice (Figure 1A,B).

**Figure 2:**
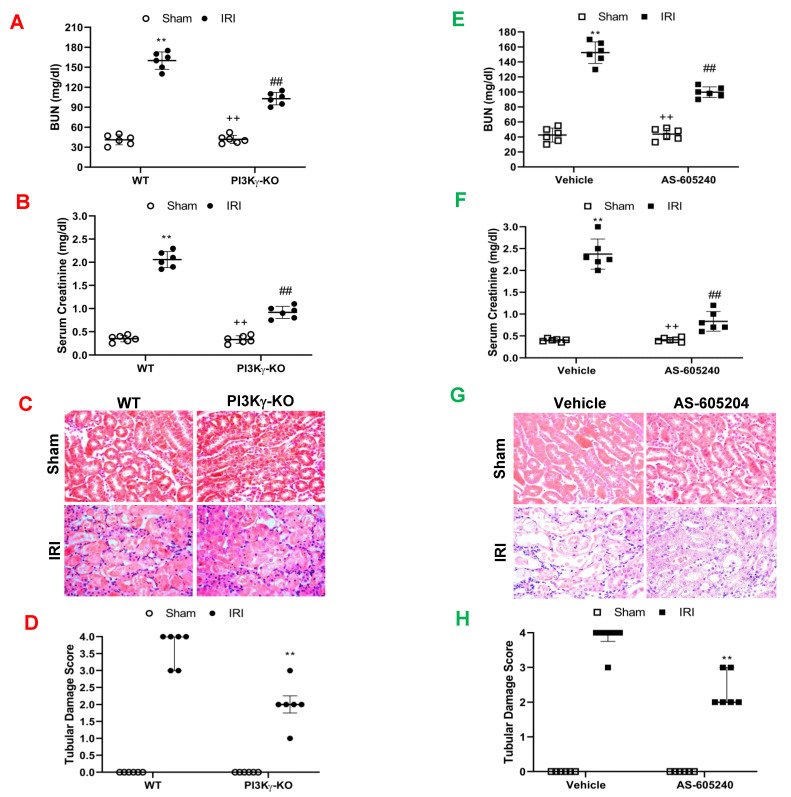
PI3Kγ deficiency or inhibition protects kidney from IRI. (**A**) Effect of PI3Kγ deficiency on blood urea nitrogen (BUN) in WT and PI3Kγ-KO mice after IRI or sham injury. ** *p* < 0.01 vs. WT sham; ^++^ *p* < 0.01 vs. PI3Kγ-KO IRI; ^##^ *p* < 0.01 vs. WT IRI *n* = 6 per group. Mean ± SD is 41 ± 7.5 for Sham in WT, 160 ± 13 for IRI in WT, 41.7 ± 6.0 for Sham in PI3Kγ, and 102.8 ± 9.4 for IRI in PI3Kγ. *n* = 6 per group. (**B**) Effect of PI3Kγ deficiency on serum creatinine in WT and PI3Kγ-KO mice after IRI or sham injury. ^##^ *p* < 0.01 vs. WT IRI; ** *p* < 0.01 vs. WT sham; ^++^ *p* < 0.01 vs. PI3Kγ-KO IRI. *n* = 6 per group. Mean ± SD is 0.35 ± 0.07 for Sham in WT, 2.05 ± 0.17 for IRI in WT, 0.33 ± 0.08 for Sham in PI3Kγ, and 0.92 ± 0.13 for IRI in PI3Kγ. *n* = 6 per group. (**C)** Representative photomicrographs of hematoxylin and eosin staining in kidney sections in WT and PI3Kγ-KO mice subjected to sham or IRI operation. (**D**) Quantitative histological assessment of tubular damage from IRI in WT and PI3Kγ-KO mice. ** *p* < 0.01 vs. WT IRI. Median with 25th and 75th percentiles is 0(0, 0) for Sham in WT, 3(2, 4) for IRI in WT, 0(0, 0) for Sham in PI3Kγ, and 1.5(1, 2) for IRI in PI3Kγ. *n* = 6 per group. (**E**) AS-605240 decrease serum BUN concentration in mice after IRI. ^##^ *p* < 0.05 vs. vehicle IRI; ** *p* < 0.01 vs. vehicle sham; ^++^ *p* < 0.05 vs. AS-605240 IRI. *n* = 6 per group. Mean ± SD is 42.50 ± 9.35 for Sham in Vehicle group, 152.50 ± 14.40 for IRI in Vehicle group, 43.67 ± 7.50 for Sham in AS-605240 group, and 99.67 ± 7.12 for IRI in AS-605240 group. (**F**) AS-605240 reduced serum creatinine in mice after IRI. ^##^ *p* < 0.05 vs. vehicle IRI; ** *p* < 0.01 vs. vehicle sham; ^++^ *p* < 0.05 vs. AS-605240 IRI. *n* = 6 per group. Mean ± SD is 0.40 ± 0.03 for Sham in Vehicle group, 2.38 ± 0.35 for IRI in Vehicle group, 0.41 ± 0.05 for Sham in AS-605240 group, and 0.83 ± 0.23 for IRI in AS-605240 group. (**G**) Representative photomicrographs of hematoxylin and eosin staining for kidney slides from the mice treated with IRI or sham injury in the presence of vehicle or AS-605240. (**H**) Tubular damage was quantitatively analyzed in the mice after IRI in the presence of vehicle or AS-605240. ** *p* < 0.05 vs. IRI. *n* = 6 in each group. Median with 25th and 75th percentiles is 0(0, 0) for Sham in Vehicle group, 3(2, 4) for IRI in Vehicle group, 0(0, 0) for Sham in AS-605240 group, and 1.5(1, 2) for IRI in AS-605240 group.

Blood urea nitrogen (BUN) and serum creatinine were significantly increased in WT mice following IRI, which indicates the IRI caused kidney dysfunction. In contrast, BUN and serum creatinine were markedly reduced in PI3Kγ KO mice (Figure 2A,B). These results indicate that kidney function is preserved in PI3Kγ KO mice. Furthermore, PI3Kγ-KO mice with IRI displayed less histological damage of the kidney, including less tubular injury, intra-tubular cast formation, and tubular dilation in comparison with WT mice (Figure 2C,D).

PI3Kγ inhibitor, AS-605240 (30 mg/kg body weight) or vehicle was used to examine if PI3Kγ can be targeted to treat kidney IRI. WT mice were subjected to 30 min ischemia followed by 24 h reperfusion. Serum BUN and creatinine were significantly increased in WT mice after IRI. In contrast, BUN and serum creatinine were considerably decreased in AS-605240-treated mice when compared with vehicle-treated mice after IRI (Figure 2E,F). Moreover, there was a significant decrease in kidney damage such as less tubular dilation, tubular injury, and intra-tubular cast formation in AS-605240-treated mice after IRI (Figure 2G,H). These results suggest that kidney function and morphology are preserved in mice treated with AS-605240 after IRI.

### 3.2. PI3kγ Deficiency Inhibits Apoptosis of Tubular Epithelial Cell after IRI

**Figure 3:**
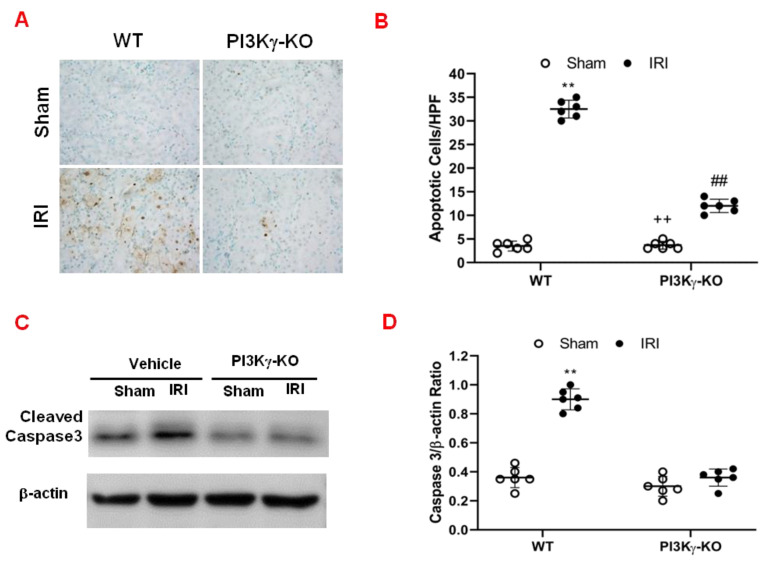
PI3Kγ deficiency protects tubular epithelial cells from apoptosis in IRI kidney. (**A**) Representative photomicrographs of kidney slides with apoptotic cells (brown) counterstaining with methyl green (green) in the IRI kidneys in WT and PI3Kγ-KO -treated mice (original magnification is 400×). (**B**) The apoptotic cells were quantitatively analyzed in the kidneys of WT and PI3Kγ-KO mice after sham injury or IRI. ^##^ *p* < 0.01 vs. WT IRI; ** *p* < 0.01 vs. WT sham; ^++^ *p* < 0.01 vs. PI3Kγ-KO IRI. Mean ± SD is 3.50 ± 1.05 for Sham in WT, 32.50 ± 1.87 for IRI in WT, 3.67 ± 0.82 for Sham in PI3Kγ, and 12.00 ± 1.41 for IRI in PI3Kγ. *n* = 6 per group. HPF means high power field. (**C**) Representative Western blot shows the expression of active caspase 3 in the kidneys in WT and PI3Kγ KO mice. (**D**) The active caspase 3 expression was quantitatively analyzed in the kidneys in WT and PI3Kγ KO mice. ** *p* < 0.01 vs. WT sham. Mean ± SD is 1.00 ± 0.14 for Sham in WT, 3.60 ± 0.44 for IRI in WT, 1.05 ± 0.48 for Sham in PI3Kγ, and 1.79 ± 1.13 for IRI in PI3Kγ. *n* = 6 per group.

We performed TUNEL-staining to determine the effect of PI3Kγ deficiency on tubular epithelial cell in response to IRI. The results demonstrated that the number of tubular apoptotic cells was significantly increased in the kidney of WT mice after IRI. However, the number of tubular apoptotic cells was markedly decreased in IRI kidney of KO mice (Figure 3A,B). These data indicate that PI3Kγ deficiency inhibits apoptotic cell death in the kidney following IRI. Caspase 3 is the final effector caspase that results in apoptotic cell death [23,24]. Therefore, the effect of PI3Kγ deficiency on caspase 3 protein expression was examined in the kidney. Western blotting analysis using antibody against active caspase 3 showed that the expression level of active caspase 3 was significantly increased in the kidney of WT mice with IRI when compared with sham-operated mice (Figure 3C and Supplementary Materials). In comparison, the level of active caspase 3 was markedly decreased in the kidney of PI3Kγ-KO mice after IRI (Figure 3C,D). These data suggest that PI3Kγ deficiency reduces caspase 3 activation in the kidney after IRI.

### 3.3. PI3kγ Deficiency or Inhibition Suppresses Infiltration of Inflammatory Cells into the Kidney

**Figure 4:**
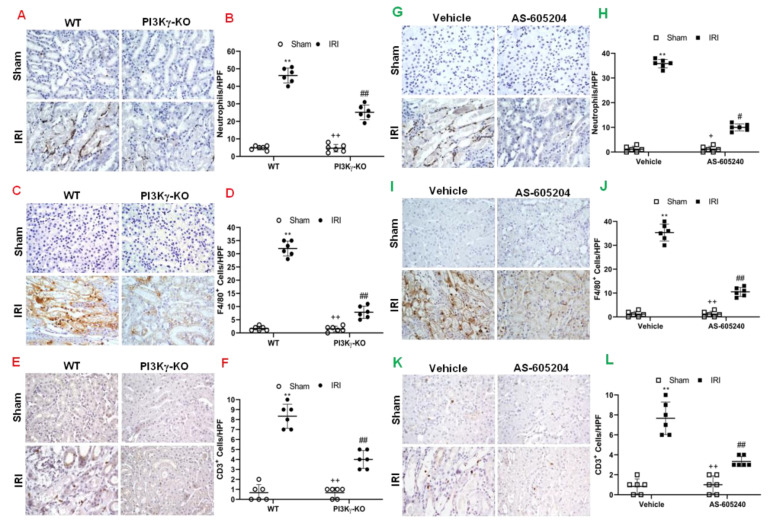
PI3Kγ deficiency or inhibition decreases infiltration of inflammatory cells in the kidney after IRI. (**A**) Representative photomicrographs of kidney slides with MPO positive (a neutrophil marker) (brown) and counterstaining with hematoxylin (blue) in PI3Kγ-KO and WT mice after sham injury or IRI. (**B**) MPO^+^ neutrophils in the kidneys were quantitatively analyzed in WT and PI3Kγ-KO mice after sham injury or IRI. ^##^ *p* < 0.01 vs. WT IRI; ** *p* < 0.01 vs. WT sham, ^++^ *p* < 0.01 vs. PI3Kγ-KO IRI. *n* = 6 per group. Mean ± SD is 4.83 ± 1.17 for Sham in WT, 45.17 ± 4.26 for IRI in WT, 4.67 ± 2.16 for Sham in PI3Kγ, and 25.17 ± 4.17 for IRI in PI3Kγ. (**C**) Representative photomicrographs of kidney slides with F4/80 (a macrophage marker) (brown) positive, nuclei were counterstained with hematoxylin (blue) in WT and PI3Kγ-KO mice after IRI or sham injury. (**D**) F4/80^+^ macrophages were quantitatively analyzed in the kidneys in WT and PI3Kγ-KO mice after IRI or sham injury. ^##^ *p* < 0.01 vs. WT IRI; ** *p* < 0.01 vs. WT sham; ^++^ *p* < 0.01 vs. PI3Kγ-KO IRI. *n* = 6 per group. Mean ± SD is 1.67 ± 0.81 for Sham in WT, 32.00 ± 2.83 for IRI in WT, 1.50 ± 1.05 for Sham in PI3Kγ, and 7.83 ± 2.32 for IRI in PI3Kγ. (**E**) Representative photomicrographs of kidney slides with CD3 (a T-lymphocyte marker) (brown) positive in WT and PI3Kγ-KO mice after IRI or sham injury. Nuclei were counterstained with hematoxylin (blue) I. (**F**) CD3^+^ T cells were quantitatively analyzed in the kidneys of WT and PI3Kγ-KO mice after IRI or sham injury. ** *p* < 0.01 vs. WT sham; ^##^ *p* < 0.05 vs. WT IRI; ^++^ *p* < 0.05 vs. PI3Kγ-KO IRI. *n* = 6 per group. Mean ± SD is 0.67 ± 0.82 for Sham in WT, 8.33 ± 1.21 for IRI in WT, 0.67 ± 0.52 for Sham in PI3Kγ, and 4.00 ± 0.89 for IRI in PI3Kγ. HPF means high power field. (**G**) Representative photomicrographs of kidney slices with MPO (brown) positive in the mice treated with sham or IRI in the presence of vehicle or AS-605240. Nuclei were counterstained with hematoxylin (blue). (**H**) MPO^+^ neutrophils were quantitatively analyzed in the kidneys of mice with sham injury or IRI in the presence of vehicle or AS-605240. ** *p* < 0.01 vs. vehicle sham, ^#^ *p* < 0.05 vs. vehicle IRI; ^+^ *p* < 0.05 vs. AS-605240 IRI. *n* = 6 per group. Mean ± SD is 1.17 ± 1.17 for Sham in Vehicle group, 35.83 ± 1.72 for IRI in Vehicle group, 1.17 ± 1.16 for Sham in AS-605,240 group, and 10.00 ± 1.41 for IRI in AS-605240 group. (**I**) Representative photomicrographs of kidney with F4/80 (brown) positive in the mice treated with sham or IRI in the presence of vehicle or AS-605240. Nuclei were counterstained with hematoxylin (blue). (**J**) F4/80+ macrophages were quantitatively analyzed in the kidneys of mice treated with sham injury or IRI in the presence of vehicle or AS-605240. ^##^ *p* < 0.05 vs. vehicle IRI; ** *p* < 0.01 vs. vehicle sham; ^++^ *p* < 0.05 vs. AS-605240 IRI. *n* = 6 per group. Mean ± SD is 1.16 ± 1.16 for Sham in Vehicle group, 35.33 ± 3.56 for IRI in Vehicle group, 1.17 ± 1.10 for Sham in AS-605240 group, and 10.50 ± 1.87 for IRI in AS-605240 group. (**K**) Representative photomicrographs of kidney slides with CD3 (brown) expression. Nuclei were counterstained with hematoxylin (blue) in the kidneys of mice treated sham injury or IRI in the presence of vehicle or AS-605240. (**L**) CD3+ T cells were quantitatively analyzed in the kidneys of mice treated with sham injury or IRI in the presence of vehicle or AS-605240. ** *p* < 0.01 vs. vehicle sham, ^##^ *p* < 0.05 vs. vehicle IRI; ^++^ *p* < 0.05 vs. AS-605240 IRI. *n* = 6 per group. Mean ± SD is 0.83 ± 0.75 for Sham in Vehicle group, 7.67 ± 1.63 for IRI in Vehicle group, 1.00 ± 0.89 for Sham in AS-605,240 group, and 3.33 ± 0.52 for IRI in AS-605240 group. HPF means high power field.

Inflammatory cells contribute to the pathogenesis of kidney IRI [25]. We examined the role of PI3Kγ in inflammatory cell infiltration into the kidney in response to IRI. We performed immunostaining of kidney sections for neutrophils, macrophages and T cells using antibodies against MPO, F4/80, and CD3, respectively. The number of neutrophils in the IRI kidney of WT mice was significantly increased. In contrast, the number of neutrophils in the IRI kidney of PI3Kγ KO mice was significantly decreased (Figure 4A,B). Moreover, a greater number of infiltrated macrophages and T cells were observed in the IRI kidney of WT mice than sham-operated control mice. In comparison, PI3Kγ deficiency markedly attenuated macrophage and T-cell infiltration into the kidney after IRI (Figure 4C–F). These results suggest that PI3Kγ functions to promote inflammatory cell infiltration into the kidney during IRI. 

We also examined the effect of PI3Kγ inhibition with AS-605240 on the inflammatory cell infiltration into the kidney. The number of neutrophils, macrophages, and T cells was significantly decreased in the IRI kidney of vehicle-treated mice with IRI when compared with sham-operated mice. In contrast, AS-605240 treatment significantly decreased the number of infiltrated neutrophils, macrophages and T-cells in the kidney after IRI (Figure 4G–L). These results suggest that AS-605240 inhibits infiltration of inflammatory cells into the kidney during IRI. 

**Figure 5:**
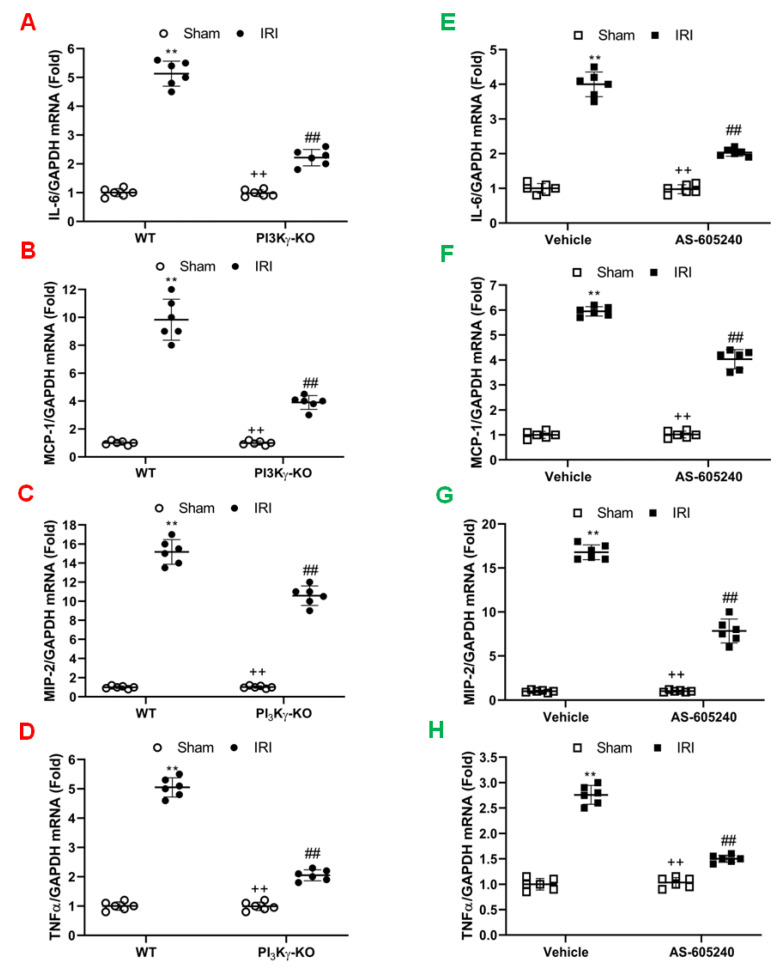
Effect of PI3Kγ deficiency or inhibition on inflammatory molecules expression in the IRI kidney. (**A**) IL-6 mRNA expressions were shown by a dot plot. ^##^ *p* < 0.05 vs. WT IRI; ** *p* < 0.01 vs. WT sham; ^++^ *p* < 0.05 vs. PI3Kγ-KO IRI. *n* = 6 per group. Mean ± SD is 1.00 ± 0.14 for Sham in WT, 5.13 ± 0.44 for IRI in WT, 0.98 ±0.12 for Sham in PI3Kγ, and 2.22 ± 0.28 for IRI in PI3Kγ. (**B**) MCP-1 mRNA expressions were shown by a dot plot. ^##^ *p* < 0.05 vs. WT IRI; ** *p* < 0.01 vs. WT sham; ^++^ *p* < 0.05 vs. PI3Kγ-KO IRI. *n* = 6 per group. Mean ± SD is 1.00 ± 0.14 for Sham in WT, 9.83 ± 1.47 for IRI in WT, 0.99 ± 0.14 for Sham in PI3Kγ, and 3.90 ± 0.50 for IRI in PI3Kγ. (**C**). MIP-2 mRNA expressions were shown by a dot plot. ^##^ *p* < 0.05 vs. WT IRI; ** *p* < 0.01 vs. WT sham; ^++^ *p* < 0.05 vs. PI3Kγ-KO IRI. *n* = 6 per group. Mean ± SD is 1.00 ± 0.14 for Sham in WT, 15.17 ± 1.29 for IRI in WT, 1.03 ± 0.13 for Sham in PI3Kγ, and 10.58 ± 1.02 for IRI in PI3Kγ. (**D**) TNFα mRNA expressions were shown by a dot plot. ^##^ *p* < 0.05 vs. WT IRI; ** *p* < 0.01 vs. WT sham; ^++^ *p* < 0.05 vs. PI3Kγ-KO IRI. *n* = 6 per group. Mean ± SD is 1.00 ± 0.15 for Sham in WT, 5.05 ± 0.33 for IRI in WT, 0.99 ± 0.14 for Sham in PI3Kγ, and 2.05 ± 0.19 for IRI in PI3Kγ. Each dot represents the kidneys from WT and PI3Kγ-KO mice after IRI or sham injury. (**E**) IL-6 mRNA expressions were shown by a dot plot. Each dot represents the kidneys from the mice treated with sham injury or IRI in the presence of vehicle or AS-605240. ** *p* < 0.01 vs. vehicle sham, ^##^ *p* < 0.05 vs. vehicle IRI; ^++^ *p* < 0.05 vs. AS-605240 IRI. *n* = 6 in each group. Mean ± SD is 1.00 ± 0.10for Sham in Vehicle group, 4.00 ± 0.35 for IRI in Vehicle group, 0.97 ± 0.13 for Sham in AS-605240 group, and 2.03 ± 0.11 for IRI in AS-605240 group. (**F**) MCP-1 mRNA expressions were shown by a dot plot. Each dot represents the kidneys from the mice treated with sham injury or IRI in the presence of vehicle or AS-605240. ** *p* < 0.01 vs. vehicle sham, ^##^ *p* < 0.05 vs. vehicle IRI; ^++^ *p* < 0.05 vs. AS-605240 IRI. *n* = 6 in each group. Mean ± SD is 1.00 ± 0.14 for Sham in Vehicle group, 5.95 ± 0.19 for IRI in Vehicle group, 1.02 ± 0.14 for Sham in AS-605240 group, and 4.03 ± 0.38 for IRI in AS-605240 group. (**G**) MIP-2 mRNA expressions were shown by a dot plot. Each dot represents the kidneys from the mice treated with sham injury or IRI in the presence of vehicle or AS-605240. ** *p* < 0.01 vs vehicle sham, ^##^ *p* < 0.05 vs. vehicle IRI; ^++^ *p* < 0.05 vs. AS-605240 IRI. *n* = 6 in each group. Mean ± SD is 1.00 ± 0.12 for Sham in Vehicle group, 16.78 ± 0.84 for IRI in Vehicle group, 1.02 ± 0.12 for Sham in AS-605240 group, and 7.83 ± 1.37 for IRI in AS-605240 group. (**H**) TNF-α mRNA expressions were shown by a dot plot. Each dot represents the kidneys from the mice treated with sham injury or IRI in the presence of vehicle or AS-605240. ** *p* < 0.01 vs vehicle sham, ^#^^#^ *p* < 0.05 vs. vehicle IRI; ^++^ *p* < 0.05 vs. AS-605240 IRI. *n* = 6 in each group. Mean ± SD is 1.00 ± 0.12 for Sham in Vehicle group, 2.76 ± 0.19 for IRI in Vehicle group, 1.03 ± 0.10 for Sham in AS-605240 group, and 1.50 ± 0.07 for IRI in AS-605240 group. GAPDH means glyceraldehyde-3-phosphate dehydrogenase.

Inflammatory cells express inflammatory molecules to stimulate kidney injury [26,27,28,29]. The mRNA expression levels of interleukin 6 (IL-6), MCP-1, tumor necrosis factor (TNF)-α, and MIP-2 were significantly increased in the kidney of WT mice after IRI when compared to sham-operated control mice. In contrast, IL-6, TNF-α, MCP-1, and MIP-2 were considerably reduced in the IRI kidney of PI3Kγ-KO mice (Figure 5A–D). Our results indicate that PI3Kγ is a critical regulator in inflammatory cytokine and chemokine expression in the kidney after IRI.

We next examined the effect of AS-605240 on the expression of pro-inflammatory molecules that contribute to the pathogenesis of kidney IRI. The mRNA expression levels of IL-6, MCP-1, TNF-α, and MIP-2 in the IRI kidney were increased markedly when compared with sham-operated controls. In contrast, the mRNA expression levels of IL-6, MCP-1, TNF-α, and MIP-2 were considerably reduced in the IRI kidney of mice treated with AS-605240 (Figure 5E-H). These results suggest that AS-605240 inhibits production of proinflammatory cytokine and chemokine in the kidney during IRI.

## 4. Discussion

In this study, we have demonstrated for the first time that PI3Kγ contributes to kidney damage after IRI. We have shown that genetic disruption or pharmacological inhibition of PI3Kγ displays protective effects on the IRI kidney by inhibiting the infiltration of the inflammatory cells, decreasing pro-inflammatory molecules production and subsequently alleviating tubular epithelial cell apoptosis. These results suggest that the PI3Kγ signaling pathway contributes to the pathogenesis of kidney injury by promoting inflammatory cell infiltration into the kidney after IRI [3].

In this study, we found that PI3Kγ is mainly expressed in the interstitial cells in the kidney after IRI and co-localizes with inflammatory cells suggesting that PI3Kγ in myeloid cells contributes to the observed phenotype of kidney injury. Recent studies have showed that PI3Kγ via G protein–coupled receptors (GPCRs) stimulates PIP3 accumulation, Akt activation, and a collection of proinflammatory responses in isolated mouse and human neutrophils, such as the formation of reactive oxygen species (ROS) and increased cell movement [30]. Further, studies have shown that PI3Kγ is required for neutrophils to accumulate at sites of inflammation in vivo [31,32]. We have recently demonstrated that adiponectin can activate PI3 kinase via its G protein-coupled receptor, contributing to the inflammatory cell recruitment into the kidney after IRI [3,33]. More recently, we have reported that inhibition of PI3Kγ suppresses CXCL16-induced monocyte migration and genetic disruption of PI3Kγ inhibits angiotensin II-induced kidney injury and fibrosis via regulation of inflammatory cell migration into the kidney [16]. In the present study, we have demonstrated that genetic disruption or pharmacological inhibition of PI3Kγ suppresses inflammatory cell migration into the kidney, inhibits proinflammatory molecule production, and reduces tubular epithelial cell apoptosis in response to IRI. These data indicate that PI3Kγ plays a crucial role in the recruitment of inflammatory cells into the kidney after IRI.

Several studies have shown that inhibition or disruption of PI3Kγ can decrease inflammatory cell infiltration [13,34,35,36]. Gruen and colleagues have reported that the disruption of PI3Kγ decreases migration and activation of macrophages and neutrophils in the knee joint after antigen-induced arthritis [30]. Another study has shown that inhibition of PI3Kγ blocked the infiltration of neutrophils into peritoneal cavity in the early phase after peritoneal injection of RANTES [37]. Moreover, the inhibition of PI_3_Kγ decreases the infiltration of inflammatory cells into the lung after bleomycin-induced pulmonary fibrosis in rats [38]. Dutra and colleagues have reported that AS-605240, a PI3Kγ inhibitor, significantly attenuated TNBS-induced acute colitis by inhibiting the NF-κB signaling pathway [39]. We have shown for the first time that inhibition of PI3Kγ could protect the kidney from IRI by reducing the accumulation of inflammatory cells in the kidney. In this study, we used PI3Kγ KO mice and PI3Kγ inhibitor to demonstrate that the PI3Kγ contributes to the pathogenesis of kidney damage after IRI by facilitating the infiltration of inflammatory cells, which leads to the production of pro-inflammatory molecules and apoptosis of the tubular epithelial cells. 

Even though PI3Kγ was found to contribute to tissue injury during IRI by recruiting inflammatory cells in this study, it was recently reported that PI3K could protect H9c2 cells from hydrogen peroxide-induced apoptosis through the PI3K/Akt/nitric oxide synthase signaling pathway [40]. Moreover, several articles demonstrated that activation of the PI3K/AKT signal pathway would suppress ischemia-reperfusion-induced renal injury [12,27,41,42]. However, these studies did not determine which kind of PI3Ks take part in the process. It will be very complicated to determine the role of PI3Ks in IRI because of its diversity. 

PI3Kγ consists of two isoforms. One isoform is the p84 regulatory subunit combined with catalytic subunit P110γ and it is widely distributed. The second isoform is the p101 regulatory subunit combined with catalytic subunit P110γ and it is relatively expressed in myeloid cells. Both regulatory subunits can function only in complex with p110γ subunits [43]. The widely distributed PI3Kγ may be the isoform of p84 and p110 and the myeloid cell PI3Kγ may be the isoform containing the p101 and p110 subunits. Studies have indicated that p101 may be involved in neutrophils migration, but p84 may regulate the chemoattractant-stimulated ROS formation. In this study, we did not differentiate the functions between p84 and p101 in kidney injury induced by IRI. Further studies are needed to identify which PI3Kγ isoform is involved in the kidney damage after IRI. 

## 5. Conclusion

In summary, this study identifies the critical role of PI3Kγ in the regulation of renal inflammation and apoptosis during the development of acute ischemic kidney injury. In response to IRI, PI3Kγ mediates the recruitment of neutrophils, macrophages, and T lymphocytes into the kidney, resulting in inflammatory molecule production and tubular epithelial cell apoptosis. These results suggest that PI3Kγ may be a potential target to prevent kidney damage induced by IRI.

## Data Availability

All data is contained within the article.

## Supplementary Materials

The following supporting information can be downloaded at: https://www.mdpi.com/article/10.3390/cells11050772/s1. Representative non-cropped Western blot shows active caspase 3 expression in the kidneys of WT and PI_3_Kγ KO mice. A Cleaved caspase 3 was presented at 19/17kDa. B β-actin was presented at 42 kDa.
