# Supplementary figures and images for: Phosphoinositide 3 Kinase γ Plays a Critical Role in Acute Kidney Injury
Source: Cells. 2022 Feb 23;11(5):772. doi: 10.3390/cells11050772 (PMC8909888; doi:10.3390/cells11050772)

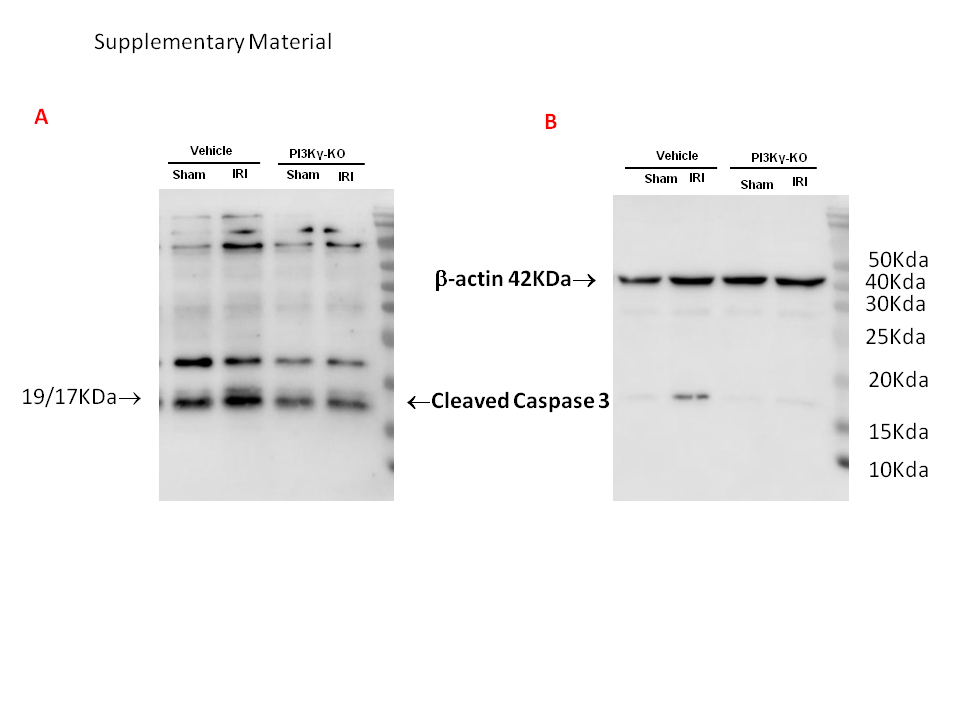

Supplement: Supplementary file 1 [file cells-11-00772-s001.zip › cells-1564825-supplementary.tif]
